# Prospective Newborn Screening for SCID in Germany: A First Analysis by the Pediatric Immunology Working Group (API)

**DOI:** 10.1007/s10875-023-01450-6

**Published:** 2023-02-27

**Authors:** Carsten Speckmann, Uta Nennstiel, Manfred Hönig, Michael H. Albert, Sujal Ghosh, Catharina Schuetz, Inken Brockow, Friederike Hörster, Tim Niehues, Stephan Ehl, Volker Wahn, Stephan Borte, Kai Lehmberg, Ulrich Baumann, Rita Beier, Renate Krüger, Shahrzad Bakhtiar, Joern-Sven Kuehl, Christian Klemann, Udo Kontny, Ursula Holzer, Andrea Meinhardt, Henner Morbach, Nora Naumann-Bartsch, Tobias Rothoeft, Alexandra Y. Kreins, E. Graham Davies, Dominik T. Schneider, Horst v. Bernuth, Thomas Klingebiel, Georg F. Hoffmann, Ansgar Schulz, Fabian Hauck

**Affiliations:** 1 Institute for Immunodeficiency, Center for Chronic Immunodeficiency (CCI), Faculty of Medicine, Medical Center - University of Freiburg, Freiburg, Germany; 2 Center for Pediatrics and Adolescent Medicine, Department of Pediatric Hematology and Oncology, Faculty of Medicine, Medical Center - University of Freiburg, Mathildenstr. 1, 79106 Freiburg, Germany; 3 Screening Center, Bavarian Health and Food Safety Authority (LGL), Oberschleissheim, Germany; 4 Department of Pediatrics, University Medical Center Ulm, Ulm, Germany; 5 Department of Pediatrics, Dr. von Hauner Children’s Hospital, University Hospital, Ludwig-Maximilians-Universität München, Munich, Germany; 6 Department of Pediatric Oncology, Hematology and Clinical Immunology, Medical Faculty, Heinrich-Heine-University - University Hospital Düsseldorf, Düsseldorf, Germany; 7 Department of Pediatrics, Medizinische Fakultät Carl Gustav Carus, Technische Universität Dresden, Dresden, Germany; 8 Center for Child and Adolescent Medicine, Heidelberg University Hospital, Heidelberg, Germany; 9 Center for Pediatrics and Adolescent Medicine, Helios Hospital Krefeld, Krefeld, Germany; 10 Department of Pediatric Respiratory Medicine, Immunology and Critical Care Medicine, Charité - Universitätsmedizin Berlin, corporate member of Freie Universität Berlin, Humboldt-Universität zu Berlin and Berlin Institute of Health (BIH), Berlin, Germany; 11 Immuno Deficiency Center Leipzig, Jeffrey Modell Diagnostic and Research Center for Primary Immunodeficiency Diseases, Hospital St. Georg, 04129 Leipzig, Germany; 12 Division of Pediatric Stem Cell Transplantation and Immunology, Clinic for Pediatric Hematology and Oncology, University Medical Center Hamburg-Eppendorf, Hamburg, Germany; 13 Pediatric Hematology and Oncology, Hannover Medical School, Hanover, Germany; 14 Division for Stem Cell Transplantation, Immunology and Intensive Care Medicine, Department for Children and Adolescents, University Hospital Frankfurt, Goethe University, Frankfurt Am Main, Germany; 15 Department for Pediatric Immunology, Rheumatology & Infectiology, Hospital for Children and Adolescents, University of Leipzig, Leipzig, Germany; 16 Division of Pediatric Hematology, Oncology and Stem Cell Transplantation, Medical Faculty, RWTH Aachen University, Aachen, Germany; 17 University Children’s Hospital, Eberhard Karls University, Tuebingen, Germany; 18 Center for Pediatrics and Adolescent Medicine, Medical Center, University Hospital Giessen, Giessen, Germany; 19 Department of Pediatrics, University Hospital of Würzburg, Würzburg, Germany; 20 Division of Pediatric Hematology and Oncology, Department of Pediatrics, University Hospital Erlangen, Erlangen, Germany; 21 Department of Pediatrics, Pediatric Intensive Care Medicine, Catholic Hospital Bochum, Ruhr-University of Bochum, 44791 Bochum, Germany; 22 Department of Immunology, Great Ormond Street Hospital for Children and UCL Great Ormond Street Institute of Child Health, London, UK; 23 Clinic of Pediatrics, Municipal Hospital Dortmund, University Witten-Herdecke, Witten, Germany; 24 Labor Berlin Charité-Vivantes, Department of Immunology, Berlin, Germany; 25 Charité - Universitätsmedizin Berlin, corporate member of Freie Universität Berlin, Humboldt-Universität zu Berlin, and Berlin Institute of Health (BIH), Berlin-Brandenburg Center for Regenerative Therapies (BCRT), Berlin, Germany; 26 Divison of Pediatric Immunology and Rheumatology, Department of Pediatrics, Dr. von Hauner Children’s Hospital, University Hospital, Ludwig-Maximilians-Universität München, Lindwurmstr. 4, 80337 Munich, Germany

**Keywords:** Severe combined immunodeficiency, SCID, Newborn screening, NBS, T cell receptor excision circles, TREC, Hematopoietic stem cell transplantation, HSCT, Thymus transplantation

## Abstract

**Backgr ound:**

T-cell receptor excision circle (TREC)-based newborn screening (NBS) for severe combined immunodeficiencies (SCID) was introduced in Germany in August 2019.

**Methods:**

Children with abnormal TREC-NBS were referred to a newly established network of Combined Immunodeficiency (CID) Clinics and Centers. The Working Group for Pediatric Immunology (API) and German Society for Newborn Screening (DGNS) performed 6-monthly surveys to assess the TREC-NBS process after 2.5 years.

**Results:**

Among 1.9 million screened newborns, 88 patients with congenital T-cell lymphocytopenia were identified (25 SCID, 17 leaky SCID/Omenn syndrome (OS)/idiopathic T-cell lymphocytopenia, and 46 syndromic disorders). A genetic diagnosis was established in 88%. Twenty-six patients underwent hematopoietic stem cell transplantation (HSCT), 23/26 within 4 months of life. Of these, 25/26 (96%) were alive at last follow-up. Two patients presented with in utero onset OS and died after birth. Five patients with syndromic disorders underwent thymus transplantation. Eight syndromic patients deceased, all from non-immunological complications. TREC-NBS missed one patient, who later presented clinically, and one tracking failure occurred after an inconclusive screening result.

**Conclusion:**

The German TREC-NBS represents the largest European SCID screening at this point. The incidence of SCID/leaky SCID/OS in Germany is approximately 1:54,000, very similar to previous observations from North American and European regions and countries where TREC-NBS was implemented. The newly founded API-CID network facilitates tracking and treatment of identified patients. Short-term HSCT outcome was excellent, but NBS and transplant registries will remain essential to evaluate the long-term outcome and to compare results across the rising numbers of TREC-NBS programs across Europe.

**Supplementary Information:**

The online version contains supplementary material available at 10.1007/s10875-023-01450-6.

## Introduction

Severe combined immunodeficiencies (SCID) are rare and life-threatening inborn errors of T-cell immunity. Most patients are asymptomatic at birth but develop severe infections and/or immune dysregulation within the first months of life. Curative treatment usually consists of hematopoietic stem cell transplantation (HSCT) or gene therapy (GT) in selected genetic entities [1, 2], but a significant minority have athymia requiring thymus transplantation. The outcome of these procedures is significantly better in patients, in whom early diagnosis and prophylactic measures prevent critical infections and end organ damage [3–5].

A real-time quantitative polymerase chain reaction (rt-qPCR) analysis for T-cell receptor excision circles (TREC) allows identification of patients with SCID, but also with other causes of severe congenital and secondary T-cell lymphocytopenia, shortly after birth. This test is performed from dried blood spots (DBS) and can be incorporated into existing newborn screening (NBS) programs [6].

Following first pilot TREC-NBS programs in Wisconsin and Massachusetts in 2008/2009, SCID was added to the Recommended Uniform Screening Panel (RUSP) of newborn screened diseases in the USA and until the end of 2018 all 50 US states were screening newborns for TREC [7, 8]. Indeed, the routine implementation of TREC-NBS had a significant impact on early diagnosis of SCID in the USA and fostered the initiation of various pilot programs across Europe [9].

NBS in Germany is regulated by the Pediatrics Directive (*Kinderrichtlinie*) of the Federal Joint Committee (*Gemeinsamer Bundesausschuss, G-BA*) [10]. This includes the NBS strategy and the reporting after abnormal NBS results. Following a political process of approximately 10 years, prospective nationwide TREC-NBS was introduced in Germany in August 2019.

This report evaluates the German TREC-NBS process and discusses remaining health political and structural challenges 2.5 years after its introduction.

## Methods

### National Implementation of a Prospective TREC-NBS in Germany

**Fig. 1:**
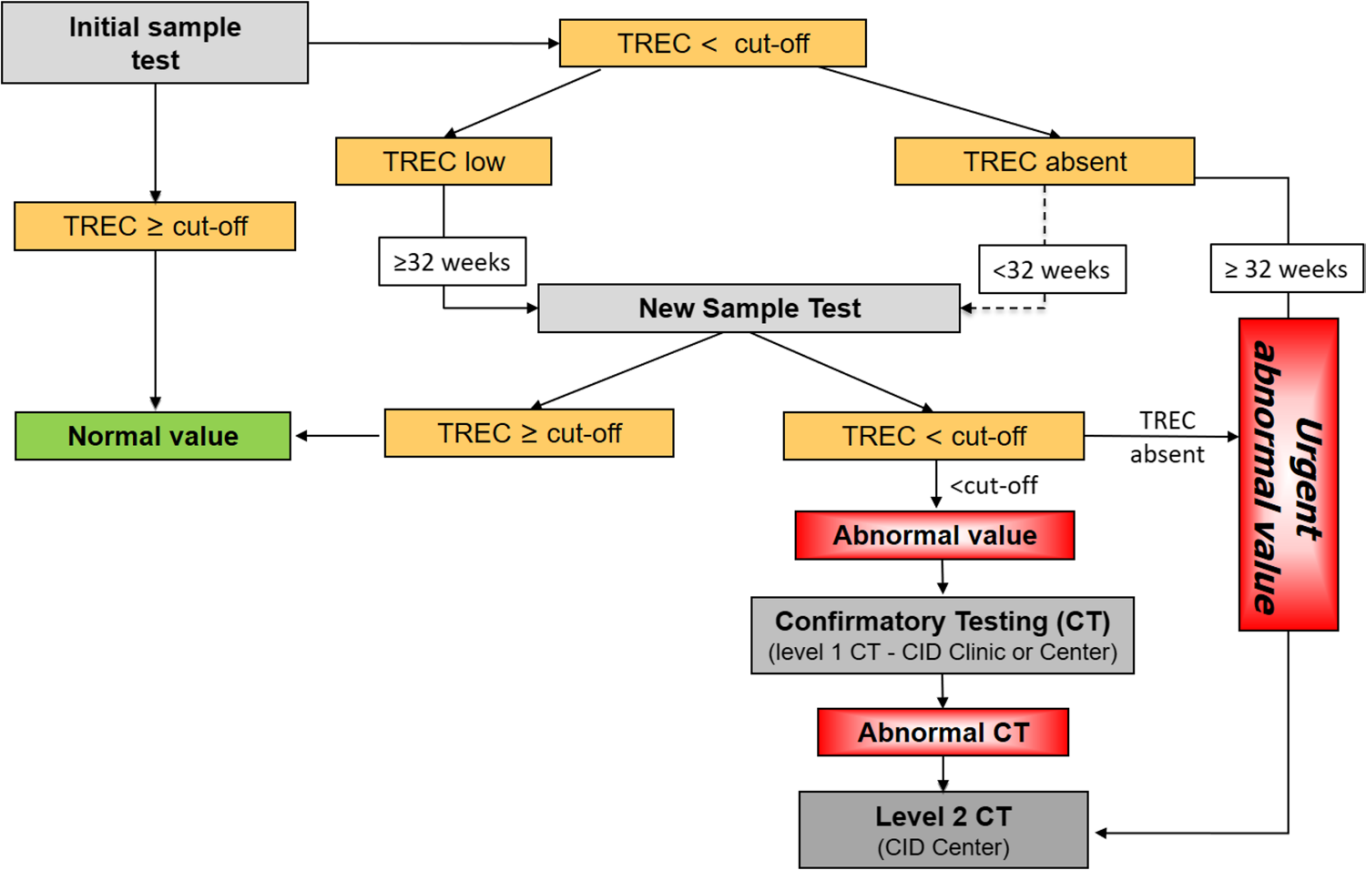
Overview of the TREC-NBS algorithm and confirmatory testing (CT) strategy. The initial sample test is performed by rt-qPCR from DBS. Both commercial and in-house protocols are used. A housekeeping gene (b-actin or RNaseP) is assessed in addition to TREC as a quality control of the test. TREC levels below the local cut-off value are classified into two categories: “TREC low” (reduced but residually detectable) and “TREC absent” (undetectable TREC level). > / < 32 weeks refers to the gestational age of the evaluated newborn. Newborns ≥ 32 weeks with “TREC absent” at initial TREC-NBS will be directly referred to a CID center for level 2 CT (“urgent abnormal value”). In patients < 32 weeks, TREC analysis will be repeated from a second card; so-called new sample test. A “new sample test” is also ordered for patients with an initial “TREC low.” In patients ≥ 32 weeks, this second analysis is performed immediately; in patients < 32 weeks of gestation, TREC-NBS is repeated at a corrected age of 32 weeks. Newborns with a confirmed “TREC low” result (“abnormal value”) will be referred to a CID clinic or center for level 1 CT. If level 1 CT is abnormal, the patient should be also evaluated by a CID center for additional diagnostic work-up (level 2 CT) and to initiate prophylactic measures and treatment. The depicted algorithm is part of the German newborn screening guideline [11]

The current algorithm of the German TREC-NBS was added to the national guideline on NBS in 2019 [11] and is summarized in Fig. 1. The nomenclature of the displayed algorithm has been adapted according to a recent recommendation for a uniform standardization of TREC-NBS terminology [12].

DBS were evaluated for TREC and for other NBS target diseases at 11 NBS laboratories (eight public and three privately run institutions). The catchment areas of the laboratories, as well as the numbers of processed samples and the federal state-lab-distributions, were variable (Figure S1). Both commercial and non-commercial TREC rt-qPCR protocols were used and the NBS laboratories are generally allowed to change or adapt platforms following their local quality control evaluations. Therefore, cut-offs for TREC levels varied and were defined by each laboratory individually.

At the initiation of TREC-NBS in August 2019, eight laboratories used commercial kits (*n* = 5 SPOT-it™, ImmunoIVD, Sweden and *n* = 3 EnLite™, PerkinElmer, Finland) and three laboratories processed samples on an in-house platform.

### Confirmatory Testing (CT) of Patients with Abnormal TREC-NBS

In accordance with the Pediatric Directive on NBS [10], reports on abnormal TREC-NBS values were relayed by the test-performing NBS laboratory to the sender of the sample, who was responsible for informing the legal guardians about the result and the recommendation for confirmatory testing (CT). The Pediatric Directive recommends that TREC-NBS CT should be carried out at specialized immunological institutions and referred the responsibility to define such institutions to the key leading medical societies [10, 13].

Following an announcement on TREC-NBS introduction in February 2019 [13], the Working Party for Pediatric Immunology (API e.v.), the German Society for Child and Adolescent Medicine (DGKJ e.V.), the German Society for Newborn Screening (DGNS), and the German Society for Pediatric Hematology and Oncology (GPOH) defined joint quality criteria for those specialized immunological institutions, termed “Combined Immunodeficiency (CID) Clinics” and “CID Centers.” CID Clinics are institutions with access to a qualified immunological diagnostic unit and experience in the initiation of prophylactic measures for (S)CID patients; CID Centers are medical institutions with additional multi-professional expertise in (S)CID diagnostics and definitive treatments. Children with “abnormal value” TREC-NBS (reduced TREC) should be referred to the nearest CID Clinic or Center for “level 1 CT.” Children with an “urgent abnormal value” TREC-NBS (absent TREC) should only be referred to a CID Center for “level 2 CT” (Fig. 1 and Table S1). Patients with confirmed T-cell lymphocytopenia and previous abnormal value TREC-NBS who underwent level 1 CT at a CID Clinic should be referred to a CID Center for in depth diagnostic evaluation, counseling, initiation of prophylactic measures, and evaluation of suitable treatment options. The list of CID Clinics and Centers has been published online [14] to facilitate patient referrals. Moreover, the API has set up a nationwide telephone hotline, where medical professionals can seek further support from pediatric immunologists. A detailed overview on the structural requirements of CID Clinics and Centers is provided in Table S1.

Genetic evaluation was initiated by the CID Clinic or Center following local diagnostic algorithms and within the regulations of the German Genetic Diagnostic Act (GenDG). Most investigations were performed by next-generation-sequencing methods, e.g., exome-based virtual panels for (S)CID-associated genes in commercial or academic laboratories.

### Analysis of Prospective Screening Data

As regulated within the Pediatric Directive on NBS (§26), the legal guardians provided written informed consent for NBS and the transfer of data to the screening laboratory [10]. Evaluation of screening-related laboratory and clinical data was performed as part of the established plausibility assessment for the German NBS program [15]. Within this assessment, primary NBS laboratory data (e.g., number of patients with a positive NBS) were evaluated in yearly intervals (DGNS report). For lack of a centralized national NBS follow-up, so-called tracking, the API performed additional 6-monthly surveys of the CID Clinics and Centers to collect clinical and CT data of patients, who eventually had a confirmed diagnosis of severe congenital T-cell lymphopenia. The regular participation in these surveys was mandatory for the CID Clinics and Centers and a dedicated API NBS working group was monitoring this process. Table 1 summarizes the clinical and laboratory core data of the API surveys; Table S2 displays the entire data set, including initial flow cytometry results of CT and family history. The CID Centers and Clinics classified the underlying cause of T-cell lymphocytopenia based on clinical, laboratory, and genetic findings and following the 2014 PIDTC diagnostic criteria for children identified by TREC-NBS [16].

**Table 1:** Clinical and laboratory core data of patients identified with severe congenital T-cell lymphocytopenia by TREC-NBS between August 2019 and February 2022

Patient no	Gestational age (weeks)	Clinical diagnosis	TREC result	Genetic diagnosis	Affected gene	Family history	HSCT	Age at HSCT (months)	Other intervention	Outcome
1	37	SCID	Absent	Yes	*IL2RG*	No	Yes	2	–	Alive and well, 27 months post Tx
2	37	SCID	Absent	Yes	*ADA*	Yes	Yes	2	ERT prior to HSCT	Alive and well, 26 months post Tx
3	39	SCID	Absent	Yes	*IL2RG*	No	No	4	–	Alive and well, 1 month post Tx
4	39	SCID	Absent	Yes	*PNP*	Yes	No	–	Chemotherapy	Deceased
5	35	SCID	Absent	Yes	*IL2RG*	Yes	Yes	1.5		Alive, sequelae from congenital CMV, 5 months post Tx
6	41	SCID	Absent	Yes	*RAG1*	No	Yes	2	–	Alive and well, 14 months post Tx
7	38	SCID	Absent	Yes	*JAK3*	No	Yes	6	–	Alive and well, 23 months post Tx
8	39	SCID	Absent	Yes	*JAK3*	No	Yes	4	–	Deceased, 6 months post Tx
9	41	SCID	Absent	Yes	*BCL11B*	No	Yes	4	–	Alive, neurological deficit (underlying disease), 24 months post Tx
10	40	SCID	Absent	Yes	*JAK3*	No	Yes	2	–	Alive and well, 16 months post Tx
11	36	SCID	Absent	Yes	*NHEJ1*	No	Yes	4	–	Alive and well, 8 months post Tx
12	39	SCID	Absent	Yes	*JAK3*	No	Yes	3	–	Alive and well, 3 months post Tx
13	38	SCID	Low	Yes	*ADA*	No	No	–	ERT, GT planned	Alive, chronic CMV infection
14	39	SCID	Absent	Yes	*DCLRE1C*	Yes	Yes	3	–	Alive and well, 27 months post Tx
15	40	SCID	Absent	Yes	*JAK3*	No	Yes	4	–	Alive and well, 18 months post Tx
16	34	SCID	Absent	Yes	*IL7RA*	No	Yes	3	–	Alive and well, 6 months post Tx
17	34	SCID	Absent	Yes	*IL7RA*	No	Yes	3	–	Alive and well, 6 months post Tx
18	37	SCID	Absent	Yes	*RAG2*	Yes	Yes	3	–	Alive and well
19	Term	SCID	Absent	Yes	*IL2RG*	No	Yes	3	–	Alive and well, 3 months post Tx
20	39	SCID	Absent	Yes	*ADA*	No	No	–	ERT, GT planned	Alive and well
21	41	SCID	Absent	No	–	No	Yes	3	–	Alive and well, 26 months post Tx
22	41	SCID	Absent	Yes	*RAG1*	No	Yes	4	–	Alive, 2 months post Tx
23	38	SCID	Absent	Yes	*IL2RG*	Yes	Yes	4	–	Alive, 2 months post Tx
24	38	SCID	Absent	Yes	*IL2RG*	No	Yes	2	–	Alive and well, 2 months post Tx
25	39	SCID	Absent	Yes	*ADA*	No	Planned	4	ERT	Alive and well
26	41	Leaky SCID	Low	Yes	*DCLRE1C*	No	Yes	4	NA	Alive and well, 24 months post Tx
27	41	Leaky SCID	Absent	Yes	*DCLRE1C*	Yes	No	–	Watch and wait	Alive and well
28	38	Leaky SCID	Low	Yes	*FOXN1 (HI)*	No	No	–	Watch and wait	Alive and well
29	39	Leaky SCID	Absent	Yes	*CORO1A*	No	Yes	5	–	Alive and well, 23 months post Tx
30	40	Leaky SCID	Absent	Yes	*DCLRE1C*	No	Yes	4	–	Alive and well, 16 months post Tx
31	41	Leaky SCID	Absent	Yes	*FOXN1 (HI)*	No	No	–	Watch and wait	Alive and well
32	41	Leaky SCID	Low	Yes	*FOXN1 (HI)*	Yes	No	–	Watch and wait	Alive and well
33	37	Omenn	Absent	Yes	*RAG1*	Yes	No	–	Immunosuppression	Deceased
34	38	Omenn	Low	Yes	*IL2RB*	No	No	–	Immunosuppression	Deceased
35	38	Omenn	Absent	Yes	*RAG1*	No	Yes	3	–	Alive and well, 23 months post Tx
36	38	ITCL	Absent	No	–	No	No	–	Watch and wait	Alive and well
37	Term	ITCL	Absent	No	–	No	No	–	Watch and wait	Alive and well
38	38	ITCL	Low	No	–	No	No	–	Watch and wait	Alive and well
39	38	ITCL	Low	No	–	No	No	–	Watch and wait	Alive and well
40	41	RTCL	Absent	No	–	No	No	–	Watch and wait	Alive and well
41	41	RTCL	Low	No	–	No	No	–	Watch and wait	Alive and well
42	40	Inconclusive	Absent	NA	NA	No	No	–	Watch and wait	Alive and well
43	39	Syndrome	Absent	Yes	22q11.2	No	No	–	Thymus Tx (2 mo)	Alive and well
44	34	Syndrome	Absent	Yes	*CHD7*	No	No	–	Watch and wait	Deceased
45	39	Syndrome	Low	Yes	*CHD7*	No	No	–	Watch and wait	Alive and well
46	38	Syndrome	Absent	Yes	*CHD7*	No	No	–	Thymus Tx (3 mo)	Alive and well
47	39	Syndrome	Low	Yes	22q11.2	No	–	–	Cardiac surgery	Alive and well
48	35	Syndrome	Low	Yes	22q11.2	No	–	–	NA	NA
49	38	Syndrome	Low	No	–	No	No	NA	Watch and wait	Alive and well
50	37	Syndrome	Absent	NA	NA	Unknown	NA	NA	Watch and wait	Deceased
51	40	Syndrome	Low	Yes	*TP63*	No	No	–	Watch and wait	Alive and well
52	38	Syndrome	Low	Yes	22q11.2	No	No	–	Watch and wait	Alive and well
53	41	Syndrome	Low	Yes	2p11.2	No	No	–	Watch and wait	Alive and well
54	Term	Syndrome	Low	Yes	Trisomy 21	No	No	–	Watch and wait	Alive, ongoing cardiological care
55	35	Syndrome	Low	Yes	*PTPN11*	No	No	–	Watch and wait	Alive and well
56	Term	Syndrome	Low	Yes	22q11.2	No	No	–	Watch and wait	Alive and well
57	37	Syndrome	Absent	Yes	*RMRP*	No	Yes	2	–	Alive and well, 23 months post Tx
58	39	Syndrome	Absent	Yes	22q11.2	No	No	–	Watch and wait	Alive and well
59	38	Syndrome	NA	Yes	22q11.2	No	No	–	Watch and wait	Alive and well
60	39	Syndrome	NA	Yes	22q11.2	No	No	–	Watch and wait	Alive and well
61	37	Syndrome	Low	Yes	*EXTL3*	No	No	–	Watch and wait	Alive and well
62	37	Syndrome	Absent	Yes	*FOXI3*	Yes	No	–	Watch and wait	Alive and well
63	41	Syndrome	Absent	Yes	22q11.2	No	No	–	Watch and wait	Deceased
64	41	Syndrome	NA	Yes	22q11.2	No	No	–	Watch and wait	Alive and well
65	35	Syndrome	NA	No	–	No	No	–	Watch and wait	Deceased
66	39	Syndrome	Absent	Yes	*RMRP*	No	Scheduled	5	–	Alive and well
67	40	Syndrome	Low	Yes	*ATM*	No	Yes	8	–	Alive and well (10 months post Tx)
68	39	Syndrome	Absent	Yes	*PAX1*	No	No	–	Thymus Tx planned	Alive and well
69	36	Syndrome	Low	No	*PPA2*	No	No	–	–	Deceased
70	37	Syndrome	Absent	Yes	22q11.2	No	No	–	Thymus Tx planned	Deceased
71	39	Syndrome	Low	Yes	22q11.2	No	No	–	Watch and wait	Alive and well
72	27	Syndrome	Absent	Yes	*RMRP*	No	No	–	Watch and wait	Alive and well
73	35	Syndrome	Absent	Yes	22q11.2	No	No	–	Thymus Tx (2 mo)	Alive and well
74	37	Syndrome	Low	Yes	22q11.2	No	No	–	Watch and wait	Alive and well
75	36	Syndrome	Absent	Yes	*RECQL4*	No	No	–	Watch and wait	Alive and well
76	38	Syndrome	Low	Yes	22q11.2	Yes	No	–	Watch and wait	Alive and well
77	39	Syndrome	Absent	Yes	22q11.2	No	No	–	Watch and wait	NA
78	30	Syndrome	Absent	Yes	Trisomy 21	No	No	–	Watch and wait	Deceased
79	38	Syndrome	Absent	Yes	*HOXA3*	Yes	No	–	Thymus Tx planned	Alive and well
80	41	Syndrome	Absent	Yes	22q11.2	No	No	–	Watch and wait	Alive and well
81	35	Syndrome	Absent	Yes	*CHD7*	No	No	–	Thymus Tx (5 mo)	Alive and well
82	38	Syndrome	Absent	Yes	22q11.2	No	No	–	Watch and wait	Alive and well
83	40	Syndrome	Low	Yes	22q11.2	No	No	–	Watch and wait	Alive and well
84	38	Syndrome	Absent	Yes	Trisomy 21	No	No	–	Watch and wait	Alive and well
85	31	Syndrome	Absent	Yes	*SGPL1*	No	No	–	Palliative care	Deceased
86	41	Syndrome	Low	Yes	*NRAS*	No	No	–	Watch and wait	Alive and well
87	40	Syndrome	Low	Yes	*TBX1*	No	No	–	Watch and wait	Alive and well
88	41	Syndrome	Low	Yes	22q11.2	No	No	–	Watch and wait	Alive and well

Along with the surveys, the CID Clinics and Centers were also asked whether they had diagnosed SCID patients, who were not identified by TREC-NBS (e.g., because of potential screening or tracking failures or in cases in whom parents had refused NBS). Due to data protection reasons, further detailed patient information, e.g., sequence details of identified genetic variants, could neither be covered by the DGNS report nor the API surveys. These extended data are part of separate and ongoing scientific evaluations within the GPOH-SCID registry (SCID-SZT 2016), which prospectively documents long-term treatment outcome of SCID patients in the periods before and after introduction of TREC-NBS [17]. Figure S2 provides an overview of the general registry structure for SCID patients in Germany and its planned extensions. A cross-check of entries between registries was not possible for data protection reasons.

Data for analyses in this report covered a period from August 2019 until December 2021 for the data of the screening laboratories (DGNS report) and August 2019 until February 2022 for CID Clinics and Centers (API surveys). Evaluation and statistical analyses of the clinical CID institutions survey data was performed using Microsoft Access 2016 and GraphPad Prism Software version 9.1.0 (221) for Windows 64-bit.

## Results

Primary screening laboratory data (DNGS report) was evaluated for a period from August 2019 until December 2021 (Figure S3 A). During this time, 1,878,985 newborns had a documented TREC-NBS, of which 1,877,057 (99.90%) had a normal value. Overall, 1443 newborns were reported with a TREC-NBS below the local cut-off value. Of these, 175 children (including 58 newborns < 32 weeks of gestation) had an urgent abnormal value in the first analyzed card. A second card (new sample test) was ordered for 1268 newborns, which was performed in 1182 (lost to follow-up, *n* = 86, 6.7%). Among these were 589 newborns with a gestational age < 32 weeks and 389 from a neonatal intensive care unit (NICU). Of 1182 new sample tests, 1022 were then reported with a normal value, 23 with an urgent abnormal value, and 137 with an abnormal value.

Documentation was incomplete to evaluate, whether all newborns with an urgent abnormal value in their original DBS were directly reported and sent for level 2 CT (as requested by the national screening algorithm, Fig. 1), or whether some laboratories (in particular during the first months after TREC-NBS introduction) instead performed a second test from a new sample. The latter seems very likely, as the number of newborns with a reported urgent abnormal TREC-NBS was significantly higher, than the documented requests for level 2 CT (Figure S3 B), but as the DGNS report data set not allowed for a cross-identification of these cases, it remains unproven.

Overall, the NBS laboratories documented 100 newborns scheduled for level 1 CT and 121 patients scheduled for level 2 CT (of which 42 were had a previously abnormal level 1 CT). Among the 100 patients requested to undergo level 1 CT, four died prior to testing (two critical ill syndromic patients from NICU and two patients without further available clinical information). Thirty-three newborns had a normal level 1 CT and therefore most likely a false positive initial TREC-NBS. The reasons for these false positive TREC-NBS remained unclear in most cases. But in some, the NBS filter card had been prepared from heparinized blood, which may cause inhibition of PCR testing [18]. For 21 patients, a secondary cause of T-cell lymphocytopenia (e.g., prematurity, gastrointestinal lymphangiectasis) was documented. Among the 121 patients with feedback reports on level 2 CT, 33 had a normal value (i.e., false positive initial TREC NBS) and 10 patients were reported to have a secondary (not further detailed) cause of T-cell lymphocytopenia. Between August 2019 and December 2021, the laboratories documented feedback reports on 78 patients with an identified primary causes of congenital T-cell lymphocytopenia (34 SCID/leaky SCID, 31 syndromic disorders, 13 other T-cell lymphocytopenia, others 10) (Figure S3). These numbers matched with the case documentation of the 6-monthly API surveys among the CID Clinics and Centers (Table 1 and Table S1).

Overall, the API surveys covered an extended observation period until February 2022 during which 10 further patients were identified, resulting in a total of 88 documented newborns with confirmed congenital T-cell lymphocytopenia between August 2019 and February 2022. Completed survey sets were for further evaluation were available in 82/88 (93%) of these newborns (Table S2).

**Fig. 2:**
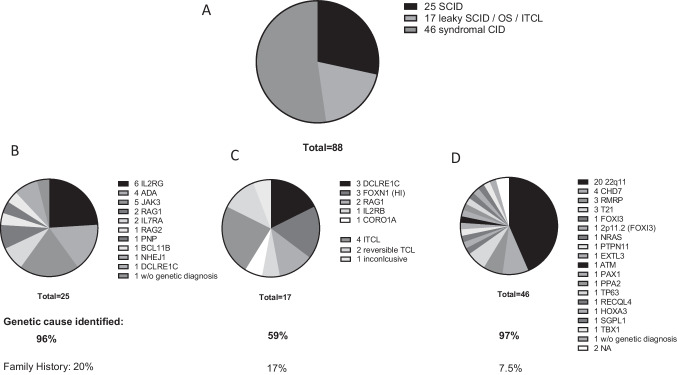
Diagnostic classification of patients following level 2 confirmatory testing at the CID centers. **A** A total of 88 patients underwent level 2 CT. Following CT evaluation (detailed in Table S2), 25 patients met diagnostic criteria for severe combined immunodeficiency (SCID); 17 patients for leaky SCID, Omenn syndrome (OS), or idiopathic T-cell lymphocytopenia (ITCL); and 46 patients for a syndromal combined immunodeficiency (CID). **B** Distribution of genetic diagnoses among the SCID patients. **C** Distribution of genetic diagnoses among the group of patients with leaky SCID. Four patients with ITCL, two patients with reversible T-cell lymphocytopenia (RTCL), and one patient with inconclusive CT finding had no genetic diagnoses. **D** Distribution of genetic diagnoses among the group of patients with a syndromal CID. Data was not available (NA) in two patients

Following the initial CT evaluations, 25/88 (28%) of patients were classified as SCID. 10/88 (11%) were diagnosed with leaky SCID or OS and 7/88 (8%) fulfilled criteria for idiopathic/reversible T-cell lymphocytopenia (ITCL/RTCL) or were judged inconclusive (*n* = 1). Furthermore, 46/88 (52%) patients had a syndromic disorder with T-cell impairment (Fig. 2A, Table 1, and Table S2).

Within the SCID group, an underlying genetic cause was reported in 24/25 patients (96%). Disease causing genetic variants in the interleukin 2 receptor subunit gamma (*IL2RG* gene) were most common (24%), followed by Janus kinase 3 (*JAK3*) (19%), adenosine deaminase (*ADA*) (15%), and recombination activating 1 or 2 (*RAG1/RAG2*) (12%). Additional four unique gene defects were detected in one case each. Details are displayed in Fig. 2B. Twenty percent of SCID patients had a positive family history of previously affected relatives (Table 1, Table S2).

Within the leaky SCID/OS group, variants in DNA cross-link repair 1C (*DCLRE1C*) (30%) and haploinsufficiency of forkhead box N1 (*FOXN1*) (30%) were the most frequently observed genetic alterations (Fig. 2C).

Despite next-generation genetic diagnostics, a genetic cause could not yet be established in 7/88 patients (8%). These are currently classified as (i) ITCL (because of persisting, often moderate, T-cell lymphocytopenia; *n* = 4), (ii) RTCL (because of normalizing T-cell counts within the first 6 months of life; *n* = 2), and (iii) undetermined cause (recently diagnosed, with further investigations pending; *n* = 1) (Fig. 2C).

Within the group of syndromic disorders with T-cell impairment, an underlying genetic cause was reported in 43/46 (93%) patients. Among these, variants impairing thymic development such as microdeletions of chromosome 22q11.2 (43%) or haploinsufficiency for chromodomain helicase DNA binding protein 7 (*CHD7*) (9%) were most frequent, followed by defects influencing the immune system and skeletal development, i.e., variants in RNA component of mitochondrial RNA processing endoribonuclease (*RMRP*) (6.5%) (Fig. 2D). Newborns with a syndromic disorder with T-cell impairment had a positive family history in 7.5% (Table 1, Table S2).

Among the 88 newborns with confirmed congenital T-cell lymphocytopenia at CT, TREC-NBS was reported with an urgent abnormal value (missing TREC) in 55 (62%) and with an abnormal value (reduced TREC) in 26 (30%). In seven (8%) newborns, details of the initial TREC results were not available. Of the 25 newborns later classified as SCID, 24 (96%) had an urgent abnormal value. One SCID patient with abnormal value TREC-NBS was diagnosed with ADA deficiency. An urgent abnormal value TREC-NBS was also reported for 21/46 (46%) newborns with a syndromic disorder, including all five infants who were later treated by thymus transplantation. Details of all reported TREC-NBS findings are summarized in Table 1 and Table S2.

In addition, the API network identified two newborns with abnormal value TREC-NBS from mothers who received immunosuppressive treatments (*n* = 1 azathioprine and *n* = 1 fingolimod) during pregnancy. At the time of CT (2 weeks of life), the lymphocyte subsets had normalized (data not shown).

**Fig. 3:**
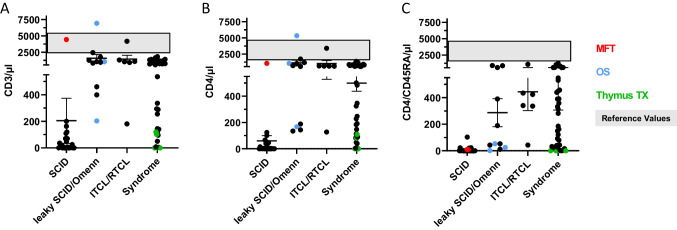
Flow cytometry results of level 1 and/or level 2 CT for patients with (urgent) abnormal TREC result. **A** Summary of CD3 + T-cell levels (/µl) in patients classified with SCID, leaky SCID/OS, ITCL/RTCL, or a syndrome with T-cell impairment. Each dot represents an individual patient. Red color indicates a patient with maternal–fetal transfusion (MFT), blue color indicates patients with OS, and green color patients who had a syndrome of T-cell impairment and eventually underwent thymus transplantation. The gray shaded area indicates the range of reference values. **B** Summary of CD4 + T-cell levels. **C** Summary of CD4 + CD45RA + T-cell levels

CT by flow cytometry confirmed severely reduced T-cell counts in 24/25 (96%) newborns classified as SCID. One SCID patient with CD3 + and CD4 + T-cell counts close to normal range for age had maternal–fetal transfusion (MFT) which was determined by short tandem repeat profiling. This patient, as well as all other SCID and OS patients, had severely reduced CD4 + CD45RA + naïve T-cells. Detailed results of CT for all 88 patients are summarized in Fig. 3A–C and Table S2.

Analysis of initial treatment decisions revealed that 86/88 (98%) reported patients were primarily treated at CID Centers. Among the 25 patients classified as SCID, 20 (80%) underwent HSCT within the first 4 months of life and two (8%) within 6 months of life. Two recently diagnosed SCID patients are scheduled for HSCT at ≤ 4 months of life. At last reported follow-up, 20/21 (95%) transplanted SCID patients were alive; the post HSCT follow-up time was 1–27 months (median 12.7). One child acquired a parainfluenza infection several weeks prior to HSCT and deceased from ARDS early after the procedure. Two patients with perinatal diagnosis of cytomegalovirus (CMV) infection survived HSCT, one with neurological sequelae. One patient with ADA-SCID receives ERT and is scheduled for GT. Furthermore, 4/10 (40%) patients classified as leaky SCID/OS (2 *DCLRE1C*, 1 RAG1, and 1 *CORO1A*) underwent HSCT within 5 months of life and all are alive (median follow-up time of 21 months). Two patients, who presented with severe OS at birth, deceased before HSCT despite early diagnosis and initiation of immunosuppressive treatment within the first 2 weeks of life. Two leaky SCID patients and four patients with ITCL (all with residual T-cell numbers and function) received prophylactic care and follow-up at CID clinics. Among the patients with syndromic disorders and T-cell impairment, 2/46 (4%) received HSCT at 7 and 8 months of age; one child with cartilage hair hypoplasia (CHH), one child with ataxia telangiectasia (AT) [19, 20], respectively. Both patients are alive 23 and 10 months after the procedure. Another patient with CHH is scheduled for HSCT within the first year of life.

Five patients with congenital athymia and complete lack of peripheral naïve T-cells underwent thymic transplantation at Great Ormond Street Hospital in London, UK. Four athymic patients, including two with 22q11.2 deletion syndrome [21] and two with CHARGE syndrome [22], were treated at ≤ 4 months of life. The fifth patient was diagnosed with a novel thymic stromal cell defect due to biallelic pathogenic variants in paired box 1 (*PAX1*), causing SCID with otofaciocervical syndrome type 2 [23] and was treated at 11 months because of persistent severe T-cell lymphopenia after a period of careful watch and wait. All patients are alive with good immune reconstitution and without evidence of immune dysregulation. Two additional patients have been diagnosed recently with athymia and have been referred for thymus transplantation.

The majority of patients with syndromic disorders and T-cell impairment (58%) are receiving supportive care and immunological monitoring. There have been eight reported deaths among these patients—all related to non-immunological and non-infectious complications (e.g., cardiac defects). Three patients with trisomy of chromosome 21 had secondary T-cell lymphocytopenia due to hydrops fetalis. All treatment decisions and outcome information are summarized in Table 1.

One patient was classified as a screening failure. This child was born at term shortly after introduction of TREC-NBS and was reported to have a normal TREC value. The child developed interstitial lung disease within the first weeks of life and was diagnosed with severe lymphocytopenia and ADA deficiency at 4 months of age. The original screening card was re-tested, which then repeatedly yielded an urgent abnormal value. The process of sample handling was discussed between the local screening laboratory and the SCID working group representatives of API and DGNS. Re-evaluation of the local procedures identified a potential technical pitfall: at the time of initial screening, the plates containing the DBS-DNA eluate were filled to high levels, thereby increasing the risk of spillage and DNA-cross-contamination between samples. The sample processing and pipetting protocol of the laboratory’s in-house PCR protocol was subsequently adapted to minimize this risk.

Another patient was classified as a tracking failure. The child was born at term and had an inconclusive TREC-NBS (absent and residually detectable TREC values in two analyses from the initial card), which should have prompted a request for a second sample. Due to lack of a NBS tracking program, it was not recognized that this second sample was never obtained. The child presented with Epstein-Barr-virus (EBV) positive lymphoma at 9 months of age and deceased shortly after from multi-organ failure. Presence of severe lymphocytopenia prompted further immunological investigations and identified PNP deficiency.

Among the 88 patients with confirmed congenital T-cell lymphocytopenia, only three patients were born < 32 weeks of gestation. All of these had syndromic disorders (CHH, trisomy of chromosome 21, and sphingosine phosphate lyase (SGPL1) insufficiency syndrome). In two of these (SGPL1 and trisomy 21), T-cell lymphocytopenia was most likely secondary due to hydrops fetalis (Table 1 and Table S2).

## Discussion

This study evaluates TREC-NBS 2.5 years after its introduction to the German NBS panel in 2019. With a birth rate of approximately 800,000/year (795,592 in 2021) and an NBS screening rate of 99.51% [15], this constitutes the largest TREC-NBS program in Europe at this point. Because of the large sample size, geographic distances, and the federal organization of the German health system, the task to implement NBS within 16 federal states is performed in 11 screening laboratories. As for other NBS target diseases in Germany, the detailed process of CT following an abnormal NBS result is not regulated within the Pediatric Directive and the G-BA delegates this responsibility to the involved medical societies [13]. This imposes significant organizational challenges and is in stark contrast to other recently initiated European TREC-NBS programs, where TREC-NBS, CT, clinical care, and follow-up are tightly regulated and usually performed in single national or regional institutions [9, 24–27].

However, the G-BA requested that CT of newborns with an abnormal TREC-NBS should be performed at specialized immunological institutions [13] and the involved medical societies defined structural and organizational requirements, which included the participation in the herein evaluated API surveys. The present comparison of these surveys with the NBS laboratory data identified consistent patient numbers, suggesting that this medical society-governed surveillance indeed resulted in a comprehensive capture of the TREC-NBS process and was able to partially compensate for the regulatory deficits concerning a NBS tracking infrastructure in Germany. To our knowledge, only two patients received CT and clinical follow-up outside the CID Clinic and Center network.

Overall, the API CID Clinics and Centers documented 88 patients with congenital and severe T-cell lymphocytopenia between August 2019 and February 2022 (25 SCID, 17 leaky SCID/OS/ITCL, and 46 syndromic disorders with T-cell impairment). The numbers of SCID patients are comparable with a clinical query of the German Surveillance Unit for rare Pediatric Diseases (ESPED) between 2014 and 2015. This previous study estimated an incidence for SCID of 1:62,500. Yet, the case definition in this former study was based primarily on clinical findings and also included some patients with syndromes (i.e., 22q11.2) and diseases, which may clinically present as SCID, but are usually not detected by TREC-NBS (e.g., ORAI1 deficiency [28]). The children of the ESPED study were mostly symptomatic at diagnosis and had a high mortality rate of 29% [29]. Our current evaluation, after introduction of prospective TREC-NBS, allowed for a more detailed evaluation of the various causes of congenital T-cell lymphocytopenia and estimated an incidence of ~ 1:54,000 for SCID, leaky SCID, and OS patients and ~ 1:41,000 for the group of syndromic disorders with T-cell impairment. The overall incidence of patients with confirmed severe congenital T-cell lymphocytopenia (including ITCL) was ~ 1:21,000. Following routine diagnostic pathways, the rate of established genetic diagnoses was very high (96% of SCID patients, 88% in all children with severe congenital T-cell lymphocytopenia), reflecting that genetic investigations for SCID and related disorders are generally well accessible within the German health system. Initial TREC-NBS series from the USA reported higher numbers of genetically unresolved (idiopathic) T-cell lymphocytopenia (ITCL) patients, of which several were later genetically diagnosed with a syndromic CID variant [30, 31].

SCID and other variants of congenital T-cell lymphocytopenia are rare diseases and detailed and long-term comparisons among the various global TREC-NBS programs, ideally in dedicated international registries, would be highly desirable. However, the continuous progress of diagnostic criteria also provides challenges. Our classification of patients followed the 2014 PIDTC criteria, which have also been the basis of previously reported and herein discussed US TREC-NBS studies [7, 16, 30, 31]. An update of this classification in November 2022 now incorporates a more detailed consideration of flow-cytometry based T-cell proliferation data [32, 33], which will allow for a more rigorous differentiation between typical, atypical, and leaky SCID cases. In the past, these data were not systematically assessed in our patient cohort and will need to be added to our prospective survey program. Nonetheless, this more detailed distinction of SCID variants seems not to imply changes of current treatment strategies [32, 33].

Interestingly, the reported incidences of SCID and further causes of severe congenital T-cell lymphocytopenia have been very similar across various North American and European regions and countries where TREC-NBS was implemented [9, 30, 31], and also the distribution of identified genetic causes was comparable with our observations. Genetic variants in the *IL2RG* gene were the most common cause reported in patients with SCID, and microdeletions of chromosome 22q11.2 were the most common genetic finding in patients with syndromic CID. Founder mutations (e.g., in *ADA* or *DCLRE1C*) and/or high rates of consanguinity have been associated with regionally higher incidences of certain SCID variants in the USA or Israel [31, 34, 35], an effect which was not observed in Germany.

While prospective multicenter long-term HSCT data of German SCID patients are collected by the GPOH SCID-SZT2016 registry [17], the API network surveys captured core information on the initial treatment decisions, including patients who were not scheduled for HSCT. Hereby we aimed (i) to evaluate the immediate impact of TREC-NBS on therapeutic management, and (ii) to identify potentially disadvantageous delays until treatment initiation. Although follow-up time was short, our results indeed indicate that the majority of identified patients had timely access to adequate treatment. This holds true for early initiation of HSCT in SCID patients, but also for disease specific treatments like ERT for ADA deficient patients, and thymus transplantation in patients with athymic syndromic disorders. With increasing numbers of TREC-NBS programs being rolled out across Europe, continuous assurance of treatment access will remain an important political challenge for the healthcare system. At present, timely access to HSCT remains problematic in some Eastern European countries, and some GT programs were stalled or have become inaccessible [36]. Although Great Ormond Street Hospital (London, UK) is the only institution with a thymus transplantation program in Europe [37], all the patients requiring this treatment received it in a timely manner given the need to confirm complete athymia and to treat and stabilize major syndromic co-morbidities prior to transplantation.

Long-term and detailed follow-up monitoring of SCID patients within the GPOH SCID-SZT 2016 registry will be important to evaluate for the effects of TREC-NBS to prevent early infections. Recent data from the USA suggest that 55% of SCID patients are experiencing relevant infections prior to HSCT despite early diagnosis by TREC-NBS [38]. In contrast, our network reported only 3/25 (12%) SCID patients with critical infections (2 patients with CMV and 1 patient with parainfluenza virus), but the survey did not systematically assess infectious presentations and the observation time was short for most patients.

Although diagnosis and initiation of treatment was achieved early for most SCID patients following TREC-NBS in Germany, our evaluation also identified the tragic case of a PNP SCID patient, who had an inconclusive TREC-NBS but did not undergo TREC-NBS re-evaluation and eventually presented clinically with fatal malignant lymphoma. This emphasizes that a systematic and federally financed follow-up strategy for all children with positive NBS results in Germany—so-called tracking—would be highly desirable. The German Pediatrics Directive on NBS does not demand a nationwide tracking strategy and only few federal states (i.e., Bavaria and Berlin-Brandenburg) have voluntarily set up routine follow-up schedules for patients with abnormal NBS results in addition to laboratory tracking. Consequently, the documented results of CT and clinical outcomes have remained insufficient for most NBS target diseases in the past [39]. Recent studies and surveys confirm this systematic deficit with missing CT follow-up information in 57% of newborns who had an abnormal NBS for cystic fibrosis and 9.3% of newborns with an abnormal NBS for metabolic disorders [15, 39, 40].

Despite structural challenges within the German NBS program, our report confirms that introduction of TREC-NBS is very successful in identifying newborns with SCID and other clinically relevant variants of congenital T-cell lymphocytopenia. The incidence of these conditions is higher than previously estimated and comparable to North America. The newly founded API-CID-network enables tracking and timely initiation of protective measures and definitive treatments of identified patients. This program initiated by a scientific medical society (API) may provide a helpful example for TREC-NBS surveillance in other countries, especially in those with federally organized healthcare systems. Nevertheless, NBS, immunodeficiency, and transplant registries will remain additional essential tools. Ideally, these registries should be accessible to all European TREC-NBS programs and use an interoperable terminology, to allow for a synchronized evaluation of the long-term screening and treatment outcome [12].

## Supplementary Information

Below is the link to the electronic supplementary material.

Supplementary file1 (DOCX 26 KB)

Supplementary file2 (XLSX 3470 KB)

Supplementary file3 (PPTX 346 KB)

Supplementary file4 (PPTX 28 KB)

Supplementary file5 (PPTX 132 KB)

## Data Availability

All data generated or analyzed during this study are included in this article.

## Abbreviations

ADA: Adenosine deaminase
API: Arbeitsgemeinschaft Pädiatrische Immunologie
CID: Combined immunodeficiency
CHARGE: Coloboma, Heart defects, Atresia choanae, Growth retardation, Genital abnormalities and Ear abnormalities
*CHD7*: Chromodomain helicase DNA binding protein 7
CHH: Cartilage-hair-hypoplasia
CMV: Cytomegalovirus
*CORO1A*: Coronin-1A
CT: Confirmatory testing
DBS: Dried blood spot
*DCLRE1C*: DNA cross-link repair 1C
DGNS: Deutsche Gesellschaft für Neugeborenenscreening
GenDG: Gendiagnostikgesetz
ERT: Enzyme replacement therapy
ESID: European Society for Immunodeficiencies
*FOXN1*: Forkhead box N1
G-BA: Gemeinsamer Bundesausschuss
GPOH: Gesellschaft für Pädiatrische Onkologie und Hämatologie
GT: Gene therapy
HSCT: Hematopoietic stem cell transplantation
*IL2RG*: Interleukin 2 receptor subunit gamma
ITCL: Idiopathic T-cell lymphocytopenia
*JAK3*: Janus kinase 3
MFT: Maternal-fetal transfusion
NBS: Newborn screening
NICU: Neonatal intensive care unit
OS: Omenn syndrome
*PAX1*: Paired box 1
PIDTC: Primary Immune Deficiency Treatment Consortium
*RAG1/2*: Recombination activating gene 1 or 2
*RMRP*: RNA component of mitochondrial RNA processing endoribonuclease
RTCL: Reversible T-cell lymphocytopenia
rt-qPCR: Real-time quantitative polymerase chain reaction
RUSP: Recommended Uniform Screening Panel
SCID: Severe combined immunodeficiency
*SGPL1*: Sphingosine phosphate lyase 1
TREC: T-cell receptor excision circle
