# Supplementary material for: Prospective Newborn Screening for SCID in Germany: A First Analysis by the Pediatric Immunology Working Group (API)
Source: J Clin Immunol. 2023 Feb 27;43(5):965–78. doi: 10.1007/s10875-023-01450-6 (PMC9968632; doi:10.1007/s10875-023-01450-6)
Supplement: Supplementary file 1 — Supplementary file1 (DOCX 26 KB) [file 10875_2023_1450_MOESM1_ESM.docx]

**Supplementary Table 1.**

Structural requirements for API CID Clinics and Centers

| **CID Clinic** (performing CT in newborns with abnormal TREC-NBS) |
| --- |
| **Staff requirements** |
| - At least 2 full-time pediatricians with experience in pediatric immunology |
| - 24h phone call availability of a doctor for newborn screening labs (incl. weekends and holidays) |
| - Availability of immediate (within 24h) appointments after abnormal TREC-NBS |
| **Structural requirements** |
|  |
| - Routine access to an immunological laboratory (locally or in collaboration with another institution, overnight shipment possible) |
| - Experience in performing SCID level 1 CT (see below) - Reporting of results to sender within 24h after assessment (Mo-Fr) |
| - Availability for round robin tests (immune phenotyping by flow cytometry) |
| **REGISTRY** |
| - Experience in entering patients into the ESID-/PID-NET-registry and commitment to timely include patients with pathological confirmatory testing |
| **Further obligations** |
| - Secondary referral of patients with an abnormal level 1 CT to a CID center |
| - Timely reporting of CT results to the initially testing NBS laboratory (for documentation within the DGNS registry) and 6-monthly to the API screening working group (for documentation within the API survey) |
| **Access to level 1 SCID laboratory assessment** |
| - Complete blood count |
| - IgM, IgG, IgA, IgE |
| - Quantitative immune phenotpying by flow cytometry: – T-cells (CD3 / CD4 / CD8) – naïve T-cell (CD45RA / CD45R0) – B-cells (CD19) – NK-cells (CD3 / CD16 / CD56) |

| **CID Center** (performing CT in newborns with urgent / abnormal TREC-NBS) |
| --- |
| **Staff requirements** |
| - At least 2 full-time pediatricians with experience in pediatric immunology |
| - 24h phone call availability of a doctor with experience in pediatric immunology – incl. availability for clinical evaluation (incl. weekends and holidays) |
| - Availability of immediate (within 24h) appointments after urgent / abnormal TREC screen |
| **Structural requirements** |
|  |
| - Onsite access to advanced diagnostic immunological laboratory |
| - Experience to perform SCID level 1 and 2 CT (see below) - Reporting of results to sender within 24h after assessment (Mo-Fr) |
| - Availability for round robin test (immune phenotyping by flow cytometry) |
| **REGISTRY** |
| - Active and timely inclusion of patients into the ESID-/PID-NET- SCETIDE and/or PRSZT/EBMT registries |
| **Further requirements** |
| - Availability of immediate inpatient treatment and medical isolation of patients - Onsite HSCT unit with documented treatment experience for IEI |
| - Forwarding of CT results to the initially testing NBS laboratory (for documentation within the DGNS registry) and 6-monthly to the API screening working group (for documentation within the API survey) |
| **Access to level 1 and 2 SCID laboratory assessment** |
| - Complete blood count |
| - IgM, IgG, IgA, IgE |
| - Quantitative immune phenotpying by flow cytometry: – T-cells (CD3 / CD4 / CD8) – naïve T-cell (CD45RA / CD45R0 / CCR7 or CD27) - – Recent thymic emigrants (CD4 / CD31 / CD45RA) – αβ- and γδ-T-cells (CD3 / αβTCR / γδTCR) – B-cells (CD19) – NK-cells (CD3 / CD16 / CD56)   In case of detectable T-cells:  – Assessment of maternal T-cell engraftment – T-cell proliferation (PHA and/or anti-CD3 / anti-CD3 / CD28) – TCR-Vbeta-Repertoire (i.e. in case of suspected oligoclonality, e.g., in OS)  Access to further differential SCID diagnostics: i.e. ADA/PNP metabolite and / or enzyme activity, genetics, exclusion of HIV. |
