# Supplementary material for: Prospective Newborn Screening for SCID in Germany: A First Analysis by the Pediatric Immunology Working Group (API)
Source: J Clin Immunol. 2023 Feb 27;43(5):965–78. doi: 10.1007/s10875-023-01450-6 (PMC9968632; doi:10.1007/s10875-023-01450-6)
Supplement: Supplementary file 3 — Supplementary file3 (PPTX 346 KB) [file 10875_2023_1450_MOESM3_ESM.pptx]

## Slide 1
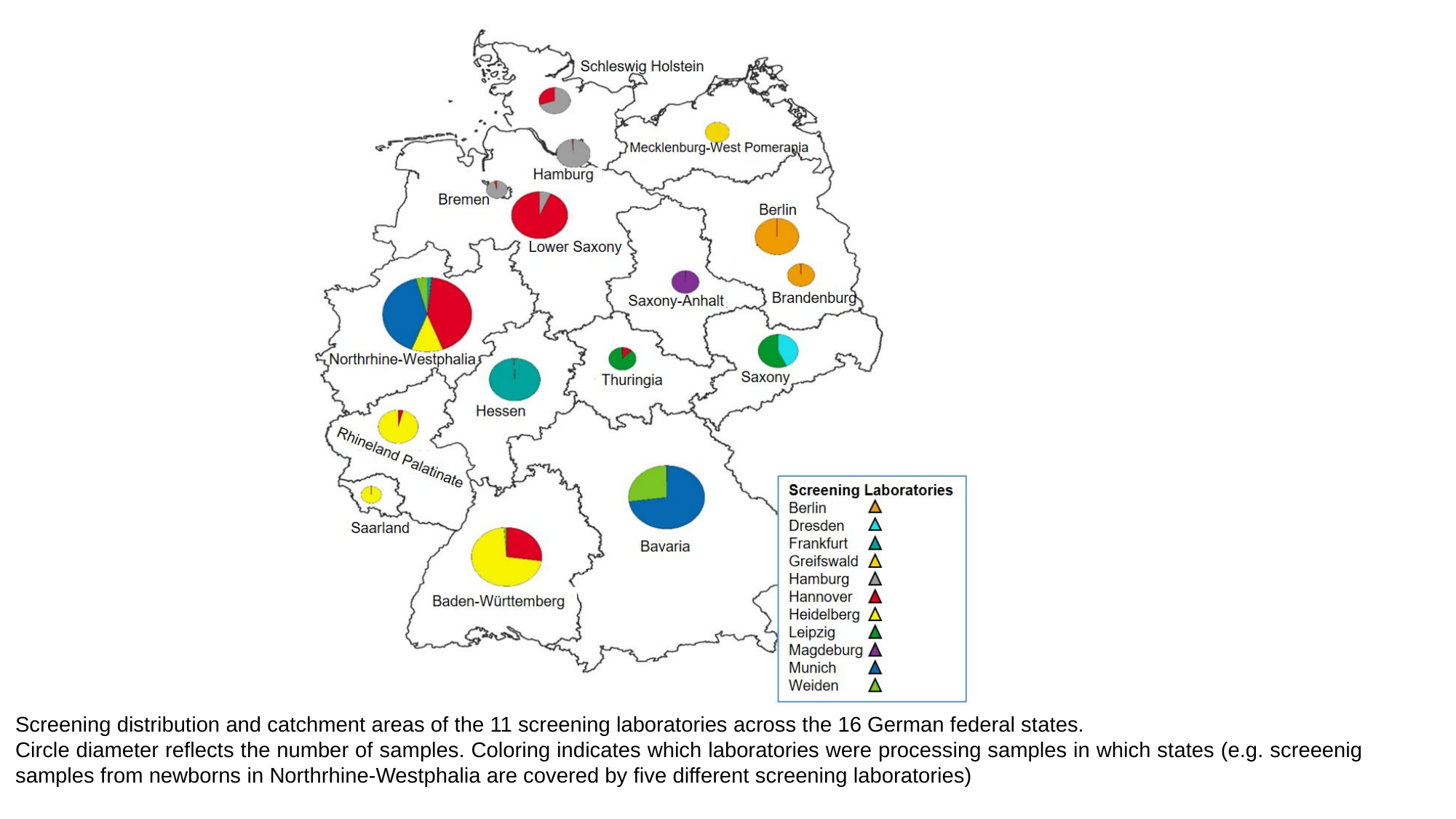

Screening distribution and catchment areas of the 11 screening laboratories across the 16 German federal states.
Circle diameter reflects the number of samples. Coloring indicates which laboratories were processing samples in which states (e.g. screeenig samples from newborns in Northrhine-Westphalia are covered by five different screening laboratories)
