# Supplementary material for: Prospective Newborn Screening for SCID in Germany: A First Analysis by the Pediatric Immunology Working Group (API)
Source: J Clin Immunol. 2023 Feb 27;43(5):965–78. doi: 10.1007/s10875-023-01450-6 (PMC9968632; doi:10.1007/s10875-023-01450-6)
Supplement: Supplementary file 4 — Supplementary file4 (PPTX 28 KB) [file 10875_2023_1450_MOESM4_ESM.pptx]

## Slide 1
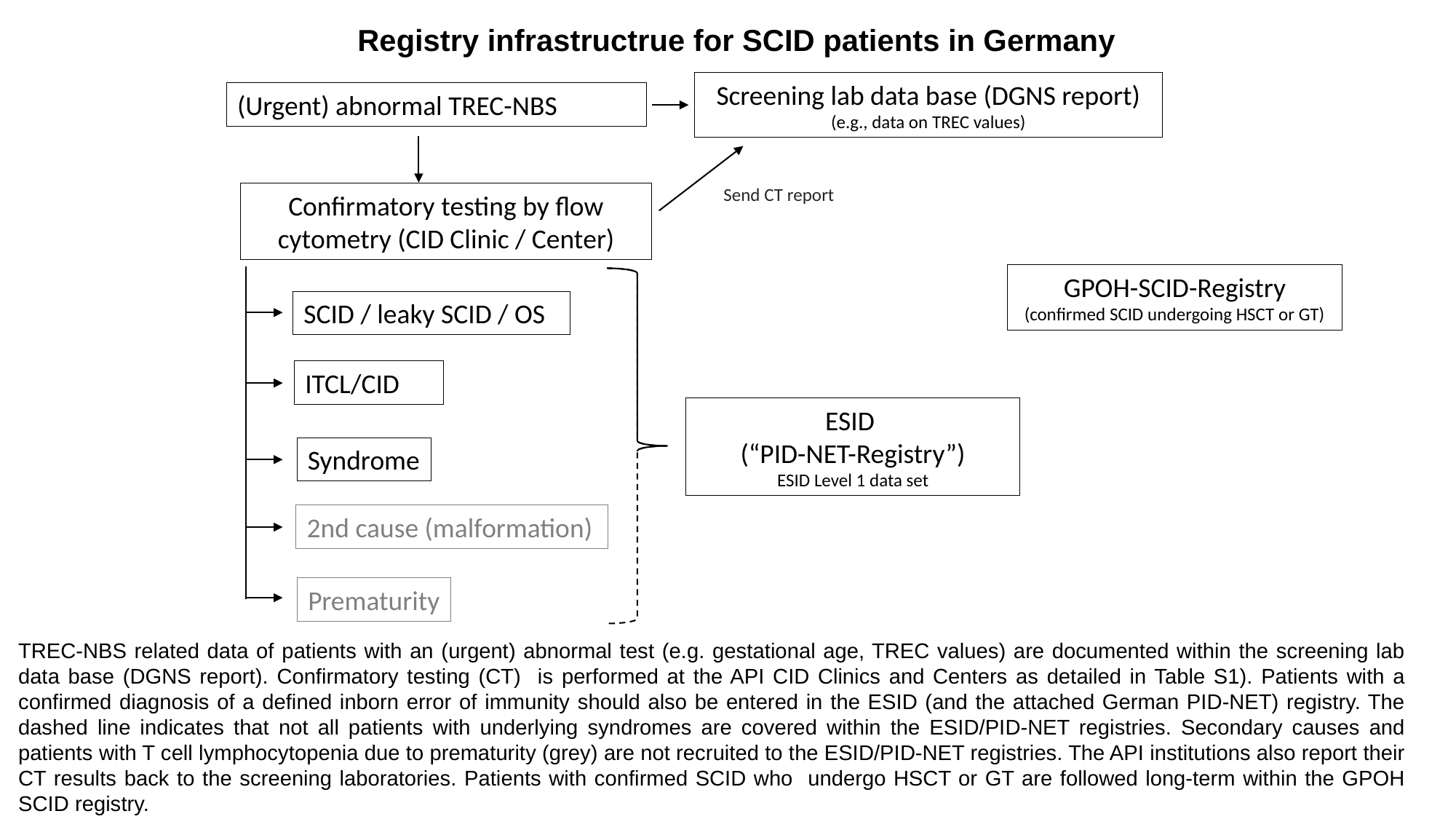

Registry infrastructrue for SCID patients in Germany
Screening lab data base (DGNS report)
(e.g., data on TREC values)
(Urgent) abnormal TREC-NBS
Send CT report
Confirmatory testing by flow cytometry (CID Clinic / Center)
GPOH-SCID-Registry
(confirmed SCID undergoing HSCT or GT)
SCID / leaky SCID / OS
ITCL/CID
ESID
(“PID-NET-Registry”)
ESID Level 1 data set
Syndrome
2nd cause (malformation)
Prematurity
TREC-NBS related data of patients with an (urgent) abnormal test (e.g. gestational age, TREC values) are documented within the screening lab data base (DGNS report). Confirmatory testing (CT) is performed at the API CID Clinics and Centers as detailed in Table S1). Patients with a confirmed diagnosis of a defined inborn error of immunity should also be entered in the ESID (and the attached German PID-NET) registry. The dashed line indicates that not all patients with underlying syndromes are covered within the ESID/PID-NET registries. Secondary causes and patients with T cell lymphocytopenia due to prematurity (grey) are not recruited to the ESID/PID-NET registries. The API institutions also report their CT results back to the screening laboratories. Patients with confirmed SCID who undergo HSCT or GT are followed long-term within the GPOH SCID registry.
