# Supplementary material for: Prospective Newborn Screening for SCID in Germany: A First Analysis by the Pediatric Immunology Working Group (API)
Source: J Clin Immunol. 2023 Feb 27;43(5):965–78. doi: 10.1007/s10875-023-01450-6 (PMC9968632; doi:10.1007/s10875-023-01450-6)
Supplement: Supplementary file 5 — Supplementary file5 (PPTX 132 KB) [file 10875_2023_1450_MOESM5_ESM.pptx]

## Slide 1
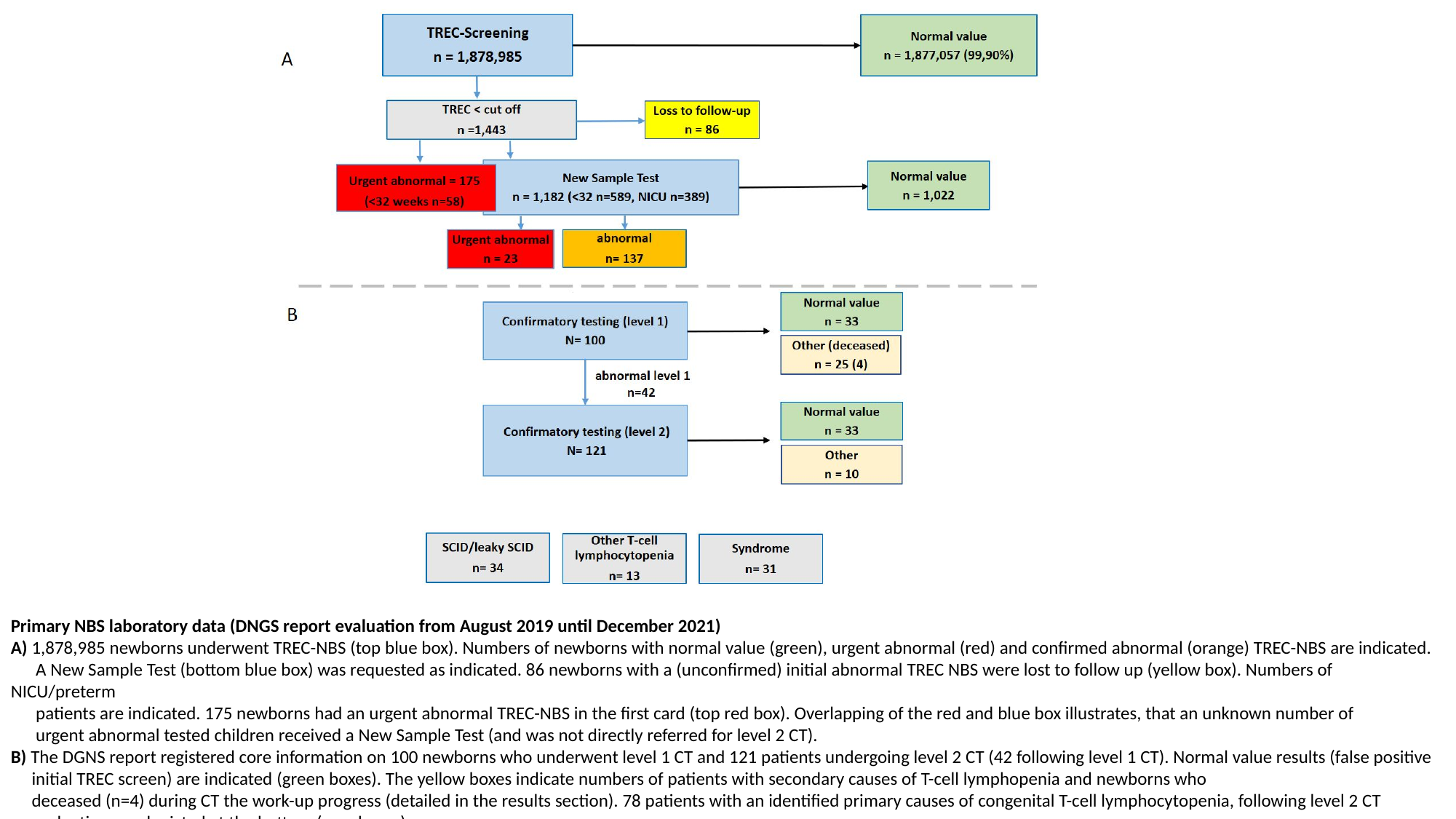

Primary NBS laboratory data (DNGS report evaluation from August 2019 until December 2021)
A) 1,878,985 newborns underwent TREC-NBS (top blue box). Numbers of newborns with normal value (green), urgent abnormal (red) and confirmed abnormal (orange) TREC-NBS are indicated.
 A New Sample Test (bottom blue box) was requested as indicated. 86 newborns with a (unconfirmed) initial abnormal TREC NBS were lost to follow up (yellow box). Numbers of NICU/preterm
 patients are indicated. 175 newborns had an urgent abnormal TREC-NBS in the first card (top red box). Overlapping of the red and blue box illustrates, that an unknown number of
 urgent abnormal tested children received a New Sample Test (and was not directly referred for level 2 CT).
B) The DGNS report registered core information on 100 newborns who underwent level 1 CT and 121 patients undergoing level 2 CT (42 following level 1 CT). Normal value results (false positive
 initial TREC screen) are indicated (green boxes). The yellow boxes indicate numbers of patients with secondary causes of T-cell lymphopenia and newborns who
 deceased (n=4) during CT the work-up progress (detailed in the results section). 78 patients with an identified primary causes of congenital T-cell lymphocytopenia, following level 2 CT
 evaluation are depicted at the bottom (grey boxes).
